# Community-based health insurance knowledge, concern, preferences, and financial planning for health care among informal sector workers in a health district of Douala, Cameroon

**DOI:** 10.11604/pamj.2013.16.17.2279

**Published:** 2013-09-17

**Authors:** Jean Jacques N Noubiap, Walburga Yvonne A Joko, Joel Marie N Obama, Jean Joel R Bigna

**Affiliations:** 1 Faculty of Medicine and Biomedical Sciences, University of Yaoundé I, Cameroon; 2 Edea Regional Hospital, Edea, Cameroon; 3 Douala General Hospital, Douala, Cameroon; 4 Bertoua Regional Hospital, Bertoua, Cameroon; 5 Goulfey District Hospital, Goulfey, Cameroon

**Keywords:** Community-based health insurance, knowledge, concern, preferences, informal sector workers, Cameroon

## Abstract

**Introduction:**

For the last two decades, promoted by many governments and international number in sub-Saharan Africa. In 2005 in Cameroon, there were only 60 Community-based health insurance (CBHI) schemes nationwide, covering less than 1% of the population. In 2006, the Cameroon government adopted a national strategy aimed at creating at least one CBHI scheme in each health district and covering at least 40% of the population with CBHI schemes by 2015. Unfortunately, there is almost no published data on the awareness and the implementation of CBHI schemes in Cameroon.

**Methods:**

Structured interviews were conducted in January 2010 with 160 informal sectors workers in the Bonassama health district (BHD) of Douala, aiming at evaluating their knowledge, concern and preferences on CBHI schemes and their financial plan to cover health costs.

**Results:**

The awareness on the existence of CHBI schemes was poor awareness schemes among these informal workers. Awareness of CBHI schemes was significantly associated with a high level of education (p = 0.0001). Only 4.4% of respondents had health insurance, and specifically 1.2% were involved in a CBHI scheme. However, 128 (86.2%) respondents thought that belonging to a CBHI scheme could facilitate their access to adequate health care, and were thus willing to be involved in CBHI schemes. Our respondents would have preferred CBHI schemes run by missionaries to CBHI schemes run by the government or people of the same ethnic group (p).

**Conclusion:**

There is a very low participation in CBHI schemes among the informal sector workers of the BHD. This is mainly due to the lack of awareness and limited knowledge on the basic concepts of a CBHI by this target population. Solidarity based community associations to which the vast majority of this target population belong are prime areas for sensitization on CBHI schemes. Hence these associations could possibly federalize to create CBHI schemes.

## Introduction

How to finance and provide health care for the more than 1.3 billion rural poor and informal sector workers in low and middle-income countries is one of the greatest challenges facing the international development community [1]. In fact, in these low income countries like Cameroon where more than 50% of the population live under the poverty threshold [2], out-of-pocket payments remain the main method of paying for health care. Unfortunately, this mode of payment limits access to quality healthcare to only the relatively rich [3, 4]. Moreover, large out-of-pocket expenditures can dramatically impoverish entire households [5]. Prepayment and sharing the burden of sickness through community-based health insurance (CBHI) have been recognized as keys for making health care affordable among the poorest [6, 7].

CBHI schemes, alternatively known as mutual health organizations are voluntary arrangements, organized at the community level. Contrary to commercial insurance organizations, they run on a non-profit basis and apply the basic principles of mutual aid and community participation in design and management [8–10]. Operating by risk-pooling, they are financed through regular premiums and are tailored to meet the needs of poorer people such as informal sector workers who would otherwise not be able to take out health insurance [9, 11–15]. They are one of the recommended financing mechanisms in the African region [16]. In many developing countries, CBHI schemes have proven to facilitate access to health care services especially among Children, pregnant women and moonlighters, a majority who are generally excluded from formal insurance [17, 18].

Since the mid-90s, promoted by many governments and international organizations, CBHI schemes have been growing in number in sub-Saharan Africa and other regions of the world [10]. In West and Central Africa, the number of CBHI schemes grew from 76 in 1997 to more than 800 by 2004 [19], and CBHI is now part of the national health financing strategy in Benin, Ghana, Rwanda, Senegal and Tanzania [20–25]. In Cameroon, in 2005, there were 60 CBHI schemes nationwide, covering less than 1% of the population [2]. In 2006, the Cameroon government adopted a national strategy aimed at creating at least one CBHI scheme in each health district and covering at least 40% of the population by CBHI schemes by 2015 [2]. Unfortunately, there is almost no published data on the implementation of CBHI in Cameroon.

Douala is the economic capital of Cameroon, and the majority of its working population is of the informal sector. This study thus aimed at evaluating CBHI knowledge, concern, preferences, and financial planning for health care among informal sector workers in a health district of Douala, Cameroon.

## Methods

### Study design and setting

This is a descriptive cross-sectional study conducted in January 2010 in the Bonassama Health District (BHD) of the Littoral region of Cameroon. The BHD covers one of the seven subdivisions of the Littoral region of Cameroon, the Douala VI subdivision. It covers an area of about 100 km2, and has a population of about 200,000 inhabitants with a very great ethnic diversity (almost all the 254 ethnic groups found in Cameroon are represented). The majority of the working population is in the informal sector. There is a CBHI scheme carrying out its activities in this health district.

### Study participants and sampling

The target population comprised of informal sectors workers of the BHD. We used an operational definition of that is similar to one that has been used in survey conducted by the International Labour Office, namely own-account (excluding administrative workers and professionals), unpaid family workers, and employers and employees working in establishments with less than 10 persons engaged [26]. To be eligible workers had to be aged 18 years or more, consenting and willing to respond to an interview. We randomly selected six of the eleven health sub-districts of the BHD: Sodiko, Bonassama, Bonaminkano, Ngwelle, Mabanda and Grand Hangar. In these selected health sub-districts, participants were recruited by convenient and consecutive sampling of eligible workers met at their jobsites. Recruitment was pursued until the required sample size of 160 was reached.

### Data collection, variables and measurements

Quantitative data was collected during face-to-face interviews using a structured pretested questionnaire containing both coded and open-ended questions. The first part of the questionnaire inquired on socioeconomic conditions such as sex, age, marital status, level of education, religion, monthly household income and household size. The second part of the questionnaire evaluated knowledge on CBHI schemes. Depending on whether or not the respondent had heard of a CBHI schemes, he was classified as “aware” or “unaware”. We only evaluated the knowledge of those who were aware of CBHI schemes. After this second stage, we lengthily explained to the respondents of both groups (“aware” or “unaware”) what exactly a CBHI scheme was. Then in the third phase, we evaluated concern and preferences pertaining to CBHI schemes. Precisely, we evaluated their willingness to be involved in a CBHI scheme, their preferences concerning key-elements of the functioning of a CBHI scheme such as the nature of members, the periodicity of paying premiums and the mean amount they were willing to pay as premiums. Finally, in the fourth part, we evaluated their practice in saving for financing health care. We used face-to-face interviews in order to avoid the exclusion of potentials participants with low levels of education and unable to read and write. The sample size of 160 was chosen by convenience.

### Data analysis

Data was coded, entered and analyzed using the Statistical Package for Social Science (SPSS) version 20.0 for Window (SPSS, Chicago, Illinois, USA). We described continuous variables using means with standard deviations, and categorical variables using frequencies and percentages. The Chi-square test or its equivalents were used to compare qualitative variables and a p value less than 0.05 was considered statistically significant.

### Ethical considerations

Before beginning this study, we obtained approval from the head of the Health Unit of the BHD. Before the interview, we obtained an informed consent from each respondent. The nature of the study, participation status, benefits of the study and confidentiality issues were made clear to the respondents before obtaining their consent.

## Results

### Socio-demographic characteristics

The socio-demographic characteristics of respondents are summarized in Table 1.

**Table 1:** Demographic characteristics of respondents

Characteristics	Number of respondents (%)
**Sex**	
Female	59 (36.9)
Male	101 (63.1)
**Age group (years)**	
18-24	27 (16.9)
25-34	74 (46.3)
35-44	45 (28.1)
≥45	14 (8.7)
**Marital status^1^**	
Single	73 (45.6)
Couple	85 (53.1)
Widowed	2 (1.3)
**Level of education^2^**	
Low	48 (30)
Middle	51 (31.9)
High	61 (38.1)
**Religion**	
None	19 (11.9)
Catholic	67 (41.9)
Protestant	59 (36.9)
Muslim	15 (9.3)
**Monthly income^3^**	
<25,000	23 (14.4)
25,000-49,999	50 (31.2)
50,000-99,999	54 (33.7)
100,000-149,999	14 (8.7)
150,000-199,999	11 (6.9)
≥200,000	8 (5)
**Monthly income/charge^3^**	
<10,000	42 (26.2)
10,000-19,999	51 (31.9)
20,000-29,999	21 (13.1)
30,000-39,999	22 (13.8)
40,000-49,999	7 (4.4)
≥50,000	17 (10.4)

1 Married or living together martially

2 Low: no or primary education. Middle: Secondary education. High: High school and University

3 CFA francs

### CBHI Knowledge

Only 25.6% (N = 41) of respondents were aware of the existence of CBHI schemes, and they were thus classified as “aware”. Among the aware group, 61% had been informed about CBHI schemes by close relatives, 14.6% through television programs, 14.6% through the radio, and 9.8% at health care centers. Awareness of CBHI schemes was significantly associated with a high level of education (p = 0.0001), but there was no significant difference between the “aware” or “unaware” groups with respect to sex, age, marital status, monthly income, and monthly income/charge (Table 2). Among the aware group, only 51.2% (N = 21) knew that there was a CBHI scheme in their town, Douala. In the aware group, 73.1% (N = 30) correctly identified CBHI schemes as an association founded on community solidarity. Eighty-five point four percent (N = 35) and 48.8% (N = 20) of respondents respectively knew the exact goal of a CBHI, and how a CBHI scheme is financed. There was no significant difference in global knowledge on CBHI scheme in the « aware group» with respect to their level of education and the channel of information on CBHI schemes (Table 3).

**Table 2:** Determinants of awareness of CBHI

Characteristics	Aware of CBHI (%)	Unaware of CBHI (%)	p-value
**Age (mean in years)**	35.6±8.2	30.8±8.6	0.2
**Gender**			
Female	14 (23.7)	45 (66.3)	0.67
Male	27 (26.7)	74 (63.3)	
**Marital status^1^**			
Single	15 (20.6)	58 (79.4)	
Couple	25 (29.4)	60 (70.6)	0.32
Widowed	1 (50)	1 (50)	
**Level of education^2^**			
Low	4 (8.3)	44 (91.7)	
Middle	11 (21.6)	40 (78.4)	<0.001
High	26 (42.6)	35 (57.4)	
**Monthly income^3^**			
<25,000	7 (30.4)	16 (69.6)	
25,000-49,999	12 (24)	38 (76)	
50,000-99,999	14 (25.9)	40 (74.1)	0.86
100,000-149,999	3 (21.4)	11 (78.6)	
150,000-199,999	4 (26.4)	7 (63.6)	
≥200,000	1 (12.5)	7 (87.5)	
**Monthly income/charge^3^**			
<10,000	10 (23.2)	32 (76.2)	
10,000-19,999	12 (23.5)	39 (76.5)	
20,000-29,999	4 (19.1)	17 (80.9)	0.44
30,000-39,999	5 (22.7)	17 (77.3)	
40,000-49,999	0 (0)	7 (100)	
≥50,000	1 (5.9)	16 (94.1)	
**Total**	41 (25.6)	119 (74.4)	

1 Married or living together martially

2 Low: no or primary education. Middle: Secondary education. High: High school and University

3 CFA francs; CBHI: Community Based Health Insurance

**Table 3:** Knowledge of the basic concepts of CBHI among the «aware» group

	Correct answer (%)	Incorrect answer (%)	p-value
**What is a CBHI scheme?**			
**Level of education**			
Low	2 (50)	2 (50)	
Middle	10 (80.9)	1 (9.1)	0.21
High	18 (69.2)	8 (30.8)	
**Channel of information**			
Television	4 (66.7)	2 (33.3)	
Radio	4 (66.7)	2 (33.3)	0.62
Hospital	4 (100)	0 (0)	
Close relatives	18 (72)	7(28)	
**What is the role of a CHBHIscheme?**			
**Level of education**			
Low	3 (75)	1 (25)	
Middle	10 (81.9)	1 (8.1)	0.73
High	22 (84.6)	4 (15.4)	
**Channel of information**			
Television	6 (100)	0 (0)	
Radio	4 (66.7)	2 (33.3)	0.32
Hospital	4 (100)	0 (0)	
Close relatives	21 (84)	4 (16)	
**Who finances a CBHI scheme?**			
**Level of education**			
Low	1 (25)	3 (75)	
Middle	4 (26.4)	7 (63.6)	0.29
High	15 (51.7)	11 (42.3)	
**Channel of information**			
Television	1 (16.7)	5 (83.3)	
Radio	3 (50)	3 (50)	0.29
Hospital	3 (75)	1 (25)	
Close relatives	13 (52)	12 (48)	

CBHI: Community-based health insurance

### CBHI concern and preferences

One hundred and thirty-eight (86.2%) respondents thought that belonging to a CBHI scheme could facilitate their access to adequate health care, and were thus willing to be involved in CBHI schemes. Those who thought that CBHI not be beneficial to them and hence did not want to be involved had a significantly low level of education (p = 0.0058). Determinants of willingness to be involved in a CBHI scheme are represented in Table 4. We asked our respondents if they preferred to belong to a CBHI scheme with members of the same ethnic group, profession or religion. The responses were “yes”, “no” or “it does not matter”. The majority of respondents declared they will belong to a CBHI scheme no matter the profession (p < 0.0001), ethnicity (p < 0.0001), or religious affiliation (p < 0.0001) of its members; what mattered most was improving their access to quality health care. We also inquired of our respondents whether or not they preferred a CBHI scheme run by the missionaries, the government or by the people of the same ethnic group. Although only 53.3% preferred management by missionaries, our respondents were significantly in favor of this management by missionaries (p < 0.0001).

**Table 4:** Determinants of willingness to be involved in a CBHI

Factors	Willing (%)	Not willing (%)	p-value
**Age (mean in years)**	32.6±7.9	29.2±6.9	0.4
Gender			
Female	53 (93.3)	6 (6.7)	0.31
Male	85 (84.2)	16 (15.8)	
**Marital status^1^**			
Single	59 (80.8)	14 (19.2)	
Couple	77 (90.6)	8 (9.4)	0.17
Widowed	2 (100)	0 (0)	
Level of education^2^			
Low	35 (72.9)	13 (27.1)	
Middle	47 (92.2)	4 (7.8)	0.0058
High	56 (91.8)	5 (8.2)	
**Monthly income^3^**			
<25,000	17 (73.9)	6 (26.1)	
25,000-49,999	44 (88)	6 (12)	
50,000-99,999	48 (88.9)	6 (11.1)	0.53
100,000-149,999	13 (92.9)	1 (7.1)	
150,000-199,999	9 (81.8)	2 (18.2)	
≥200,000	7 (87.5)	1 (12.5)	
Monthly income/charge			
<10,000	37 (88.1)	5 (11.9)	
10,000-19,999	44 (92.3)	7 (13.7)	
20,000-29,999	20 (95.2)	1 (4.8)	0.22
30,000-39,999	18 (81.8)	4 (18.2)	
40,000-49,999	4 (57.2)	3 (42.8)	
≥50,000	15 (88.2)	2 (11.8)	
**Total**	138 (86.3)	22 (13.7)	

1 Married or living together martially

2 Low: no or primary education. Middle: Secondary education. High: High school and University

3 CFA francs; CBHI: Community-based health insurance

Pertaining to the periodicity of paying premiums, 75.4% of respondents preferred monthly premiums, 14.5% quarterly premiums, 4.3% biannual premiums and 5.8% annual premiums. Those who preferred monthly premiums declared it will be a lot easier to pay monthly premiums than to pay a bulk sum once a year. The mean amount they were willing to pay as insurance premium was 704±737 CFA francs/charge (1.39±1.45 USD), with a minimum of 50 CFA francs (0.1 USD) and a maximum of 5000 CFA francs (9.84 USD).

A hundred and ten (68.7%) respondents belonged to a solidarity based community association. The majority of these people (86.9%) said they would accept to fuse their individual associations to create a CBHI scheme.

### Financial planning for health care

Only 4.4% (N = 7) of respondents had health insurance: five had commercial health insurance and only two (1.2%) belonged to a CBHI scheme. Seventy-eight (48.7%) saved for health care in savings which were not specific for health but could be used for any other necessity. Forty-seven did nothing to prepare for financial sickness burden.

A hundred and ten (68.7%) belonged to at least one solidarity based community association. The majority of these solidarity based community associations (83.5%) had an “emergency saving”. These emergency savings were obtained from obligatory monthly contributions from all members. From these emergency savings, money could be gotten to help members in times of need including sickness.

## Discussion

The objective of this study was to evaluate CBHI knowledge, concern and preferences of informal sector workers of the BHD. In addition, the study aimed at assessing their practices in financial planning for health care. We found that only 4.4% of respondents had health insurance, and specifically 1.2% were involved in a CBHI scheme. A previous evaluation in 2005 had indicated that, less than 1% of the Cameroonian population was covered by a CBHI scheme [2]. In accordance with this previous report, our finding suggests that there may still be a very low coverage of the informal sector workers in Douala and globally in Cameroon. Studies in other African countries have demonstrated that CBHI schemes have not been effective in reaching out the majority of their target populations, the poor, and the informal sector workers [27–30]. Our results show that this very low involvement of our respondents in CBHI schemes is essentially due to a low awareness and knowledge of basic concepts of CBHI.

Indeed, only 25.6% of our respondents had heard about a CBHI scheme. This suggests that only very few workers of the informal sector of the BHD were aware of the existence of CBHI schemes, although there is one in their town. In a recent study, Donfouet et al. also found a low awareness of the existence of CBHI scheme among household heads of rural communities in Cameroon [31]. This low awareness of CBHI schemes is probably due to inadequate public sensitization through the mass media. We noticed that irrespective of sex, age or level of education, close to 75% of the aware group knew that CBHI schemes were solidarity based community organizations with the main aim of facilitating access to quality health care. But on the other hand, only 48.8% of the aware group knew how a CBHI scheme was financed. Yet adequate knowledge of what a CBHI scheme is, its role and its management are necessary for adhesion to a CBHI scheme [21]. As financing is one of the corner stones of a CBHI scheme, it is of utmost importance that the population should be adequately informed on this aspect, which was not observed in our sample population. In 2006, the Cameroonian government has adopted a national strategic plan which aims at creating at least one CBHI scheme in each health district and covering at least 40% of the population by CBHI by 2015 [2]. To attain this objective, effective social marketing strategies must be implemented through information-education and communication on CBHI schemes. After haven explained to our respondents to our respondents what a CBHI scheme was, mainly its way of functioning, 86.8% of them thought that belonging to a CBHI could facilitate their access to adequate health care, and were thus willing to be involved in a CBHI scheme. This proportion is similar to those found among household heads in rural communities in North-Central Nigeria (87%), and in Cameroon (93.98%), by Babatunde et al. and Donfouet et al. respectively [31, 32]. This equally proves that when the general population is adequately informed about CBHI schemes, the vast majority wish to join a CBHI scheme in order to facilitate access to quality health care.

For several years, African countries have had to contend with absence of good governance. And this contributes to impoverish its people. This has considerably contributed to reduce the confidence level of the population in government public administration. On the contrary, the missionaries who have successfully invested in health and social projects have progressively gained the people's confidence. This is highlighted in our study by the fact that, our respondents were significantly in favor of joining a CBHI scheme run by missionaries (<0.0001). Thus adhesion to CBHI schemes would greatly be encouraged if these CBHI schemes were run by missionaries. For example, a CBHI scheme could be included directly in missionary-based hospitals with a high clientele.

Belonging to a solidarity based community association could reflects one's willingness to join a CBHI. The strong degree of community solidarity is an important factor in establishing a community health care prepayment scheme [31]. The vast majority of Cameroonians with relatively low income belong to a solidarity based community association. This is observed in our study and may facilitate adhesion to CBHI schemes. Furthermore, 86.9% of the respondents who belonged to a solidarity based community association were in favor of fusing their individual groups to create a CBHI scheme. In fact, mass communication campaigns and the education of the population in solidarity based community associations on CBHI could significantly contribute to increase adherence to CBHI schemes. Hence, new adherents to CBHI schemes could be recruited directly from solidarity based community associations. Hence there will be considerable advantages in creating and developing CBHI schemes by federalizing traditional, professional, and regional solidarity based community associations.

In our study 83.5% of solidarity based community associations to which our respondents belonged had emergency savings. These savings are obtained from small obligatory monthly contributions from all its members and from which money could be gotten to help a member in proven need, amongst which health problems. This system of assistance is similar to the principle of CBHI schemes. This suggests that the role of CBHI schemes in covering health risk through prepayment could easily be understood among solidarity based community associations.

Some relevant data obtained from this study is the premiums respondents are willing to pay for the CBHI. The mean premium was approximately 704 CFA francs (1.39 USD) per person and per month. Donfouet et al., and Ichoku et al. respectively found a mean of premiums of 2.15 USD and 1.5 USD among households head in Cameroonian and Nigerian rural communities [31, 33]. Data on the premium respondents are willing to pay for the insurance are important for health service managers and policy makers to set premiums that will not exceed the amount households can afford to pay (Table 5).

**Table 5:** Premium respondents are willing to pay for the insurance per person per month

Premium^1^	Number of respondents (%)	Mean ± Standard deviation*
<250	29 (21.32)	123,89±38,73
250-499	29 (31.32)	296,97±55,38
500-749	43 (31.62)	582,51±86,85
750-999	3 (2.21)	833±0
≥1000	32 (23.53)	1766,72±837,38

3 CFA francs

The limitation of our study is the non-random sampling of participants and the convenient sample of 160 that imply imprecision in the estimates. Further studies with larger samples are thus needed to confirm these findings.

## Conclusion

Our study reveals a very low participation in CBHI schemes among the informal sector workers of the BHD. This is mainly due to the lack of awareness and limited knowledge on the basic concepts of CBHI by this target population. Increasing adherence to CBHI schemes requires effective social marketing strategies using information-education and communication through television, radio and in hospitals and communities. Solidarity based community associations to which the vast majority of this target population belong are prime areas for sensitization on CBHI schemes. Hence these associations could federalize to create CBHI schemes. Moreover, bearing in mind the trust the population has in the missionaries, their participation in running of CBHI schemes could increase adherence, mainly by incorporating CBHI schemes in missionary hospitals. Finally, to set premiums for CBHI schemes, it is capital to take into account the mean amount the households’ heads are willing to pay which is approximately 704 CFA francs (1.39 USD) in our study.

## References

[1] Preker AS, Carrin G, Dror D, Jakab M (2002). Effectiveness of community health financing in meeting the cost of illness. Bull World Health Organ..

[2] Ministère de la Santé Publique du Cameroun (2006). Plan stratégique pour la promotion et le développement des mutuelles de santé au Cameroun.

[3] Palmer N, Mueller D, Gilson L, Mills A, Haines A (2004). Health financing to promote access in low income settings-how much do we know. Lancet..

[4] Gilson L (1997). The lessons of user fee experience in Africa. Health Policy Plan..

[5] van Doorslaer E, O'Donnell O, Rannan-Eliva RP, Somanathan A (2006). Effect of payments for health care on poverty estimates in 11 countries in Asia: an analysis of household survey data. Lancet..

[6] Jutting JP (2001). The Impact of Health Insurance on the Access to Health Care and Financial Protection in Rural Developing Countries: The Example of Senegal.

[7] Sepehri AS, Sarma W, Simpson (2006). Does non-profit health insurance reduce financial burden? Evidence from the Vietnam Living Standards Survey Panel. Health Econ..

[8] Carrin G, Waelkens MP, Criel B (2005). Community-based health insurance in developing countries: a study of its contribution to the performance of health financing systems. Trop Med Int Health..

[9] Ekman B (2004). Community-based health insurance in low-income countries: a systematic review of the evidence. Health Policy Plan..

[10] Bennett S (2004). The role of community-based health insurance within the health care financing system: a framework for analysis. Health Policy Plan..

[11] Dror DM, Jacquier C (1999). Micro-Insurance: Extending Health insurance to the Excluded. International Social Security Review..

[12] Carrin G, Waelkens M, Criel B (2005). Community-based health insurance in developing countries: a study of its contribution to the performance of health financing systems. Trop Med Int Health..

[13] Devadasan N, Ranson K, Van Damme W, Acharya A, Criel B (2006). The landscape of community health insurance in India: an overview based on 10 case studies. Health Policy..

[14] Ranson MK (2002). Reduction of catastrophic health care expenditures by a community-based health insurance scheme in Gujarat, India: current experiences and challenges. Bull World Health Organ..

[15] Ranson MK, Sinha T, Chatterjee M, Acharya A, Bhavsar A, Morris SS, Mills AJ (2006). Making health insurance work for the poor: learning from the Self-Employed Women's Association's (SEWA) community-based health insurance scheme in India. Soc Sci Med..

[16] World Health Organization (WHO) (2006). Health financing: a strategy for the African region. Regional Committee for Africa: Fifty-sixth session.

[17] Dror DM, Preker AS (2002). Social reinsurance. A new approach to sustainable community health financing.

[18] Shaw RP, Griffin SC (1995). Financing health care in Sub-Saharan Africa through user fees and insurance.

[19] Gamble-Kelley A, Diop F, Makinen M (2006). Approaches for scaling up community-based health financing schemes.

[20] De Allegri M, Sanon M, Bridges J, Sauerborn R (2006). Understanding consumers’ preferences and decision to enrol in community-based health insurance in rural West Africa. Health Policy..

[21] De Allegri M, Sanon M, Sauerborn R (2006). To enrol or not to enrol: aqualitative investigation of demand for health insurance in rural WestAfrica. Social Sci Med..

[22] Dong H, De Allegri M, Gnawali D, Souares A, Sauerborn R (2009). Drop-out analysis of community-based health insurance membership at Nouna, Burkina Faso. Health Policy..

[23] Dong H, Mugisha F, Gbangouc A, Kouyate B, Sauerborn R (2004). The feasibility of community-based health insurance in Burkina Faso. Health Policy..

[24] Franco LM, Diop FP, Burgert CR, Kelley AG (2008). Effects of mutual health organizations on use of priority health-care services in urban and rural Mali: a case-control study. Bull World Health Organ..

[25] Jütting J (2003). Health insurance for the poor? OECD: Determinants of participation in community-based health insurance schemes in rural Senegal.

[26] Hussmanns R (1998). Developments in the design and implementation of informal sector and similar surveys-a review of national practices and experiences.

[27] Mathauer I, Schmidt JO, Wenyaa M (2008). Extending social health insurance to the informal sector in Kenya. An assessment of factors affecting demand. Int J Heal Plan Manag..

[28] Kimani D, Muthaka DI, Manda DK (2004). Healthcare Financing Through Health Insurance in Kenya: The Shift to A National Social Health Insurance Scheme.

[29] Carrin G, Chris James, Michael Adelhardt, Ole Doetinchem (2007). Health financing reform in Kenya -assessing the social health insurance proposal. S Afr Med J..

[30] Kimani JK, Ettarh R, Kyobutungi C, Mberu B, Muindi K (2012). Determinants for participation in a public health insurance program among residents of urban slums in Nairobi. Kenya: results from a cross-sectional survey< BMC Health Serv Res..

[31] Donfouet HP, Makaudze E, Mahieu PA, Malin E (2011). The determinants of the willingness-to-pay for community-based prepayment scheme in rural Cameroon. Int J Health Care Finance Econ..

[32] Babatunde OA, Akande TM, Salaudeen AG, Aderibigbe SA (2012). Willingness to Pay for Community Health Insurance and its Determinants among Household Heads in Rural Communities in North-Central Nigeria. IRSSH..

[33] Ichoku EH, Fonta W, Atagbua J (2010). Estimating the willingness to pay for community health insurance schemes in Nigeria: A random valuation framework. The IUP Journal of Risk and Insurance..

